# Human rabies postexposure prophylaxis during a raccoon rabies epizootic in New York, 1993 and 1994.

**DOI:** 10.3201/eid0503.990312

**Published:** 1999

**Authors:** J D Wyatt, W H Barker, N M Bennett, C A Hanlon

**Affiliations:** University of Rochester School of Medicine & Dentistry, New York 14642, USA. jeff_wyatt@urmc.rochester.edu

## Abstract

We describe the epidemiology of human rabies postexposure prophylaxis (PEP) in four upstate New York counties during the 1st and 2nd year of a raccoon rabies epizootic. We obtained data from records of 1,173 persons whose rabies PEP was reported to local health departments in 1993 and 1994. Mean annual PEP incidence rates were highest in rural counties, in summer, and in patients 10 to 14 and 35 to 44 years of age. PEP given after bites was primarily associated with unvaccinated dogs and cats, but most (70%) was not attributable to bites. Although pet vaccination and stray animal control, which target direct exposure, remain the cornerstones of human rabies prevention, the risk for rabies by the nonbite route (e. g., raccoon saliva on pet dogs' and cats' fur) should also be considered.


### Human Rabies Postexposure Prophylaxis during a Raccoon Rabies Epizootic in New York, 1993 and 1994

**Jeffrey D. Wyatt,* William H. Barker,***
**Nancy M. Bennett,† and Cathleen A. Hanlon‡**
*University of Rochester School of Medicine & Dentistry, Rochester, New York, USA; †Monroe County Department of Health, Rochester, New York, USA; ‡Centers for Disease Control & Prevention, Atlanta, Georgia, USA

We describe the epidemiology of human rabies postexposure prophylaxis (PEP) in four upstate New York counties during the 1st and 2nd year of a raccoon rabies epizootic. We obtained data from records of 1,173 persons whose rabies PEP was reported to local health departments in 1993 and 1994. Mean annual PEP incidence rates were highest in rural counties, in summer, and in patients 10 to 14 and 35 to 44 years of age. PEP given after bites was primarily associated with unvaccinated dogs and cats, but most (70%) was not attributable to bites. Although pet vaccination and stray animal control, which target direct exposure, remain the cornerstones of human rabies prevention, the risk for rabies by the nonbite route (e.g., raccoon saliva on pet dogs' and cats' fur) should also be considered.

Raccoon rabies, present in the southeastern United States since the 1950s, became responsible for an epizootic in the U.S. mid-Atlantic region during the 1970s after raccoons were translocated there for hunting (1). The introduction of the variant of rabies virus associated with raccoons into a rabies-naive raccoon population caused the most intensive animal rabies outbreak on record, in part because of the abundance of raccoons in suburban environments throughout the mid-Atlantic and northeastern metropolitan areas. Raccoon rabies affects approximately one million square kilometers of the eastern United States with a human population of approximately 90 million.

Since the mid-Atlantic raccoon rabies epizootic entered New York State in 1990, the number of rabid animals increased from 54 in pre-epizootic 1989 to 2,746 (89% raccoons) in 1993—the largest number of rabid animals ever reported from any state (2). Despite traditional public health measures for rabies control (e.g., pet vaccination, stray animal control, public education), human rabies postexposure prophylaxis (PEP) rates inevitably increased with the arrival of the epizootic front (3). Preliminary data from New York documented a 4,000% increase in the absolute number of persons receiving PEP, from 81 (1989) to 3,336 (1993) (4). The epidemiologic trends of human PEP in New York State remain largely undescribed.

One of the Healthy People 2000 objectives formulated by the U.S. Public Health Service is to reduce by 50% the need for human rabies PEP by the year 2000 (5). A reduction in the number of PEP cases, which are not reportable, appears unattainable without first defining the numerator, as well as the epidemiologic characteristics of precipitating events leading to suspected rabies exposure and inappropriate treatments.

We describe demographic and animal exposure data associated with human rabies PEP in an area with epizootic raccoon rabies. The epidemiologic description is intended to assist medical practitioners and public health officials in reducing the incidence of human and domestic animal exposure to rabid animals and thus in minimizing the need for PEP in communities affected by the raccoon rabies epizootic.

Address for correspondence: Jeff Wyatt, University of Rochester, Box 674, Rochester, New York 14642, USA; fax: 716-273-1085; e-mail: jeff_wyatt@urmc.rochester.edu.

### The Study

### Setting

Four contiguous upstate New York counties (Monroe, Wayne, Cayuga, and Onondaga) were first affected by the raccoon rabies epizootic between December 1992 and June 1993 (Figure 1). Monroe and Onondaga Counties, encompassing the cities of Rochester and Syracuse, are predominantly urban-suburban, with human population densities of 414 per square kilometer and 230 per square kilometer, respectively. Wayne and Cayuga Counties are predominantly rural-suburban, with relatively lower population densities of 57 and 45 people per square kilometer, respectively. The four-county region in western upstate New York comprises 7,090 square kilometers and has an estimated human population of 1,354,377.

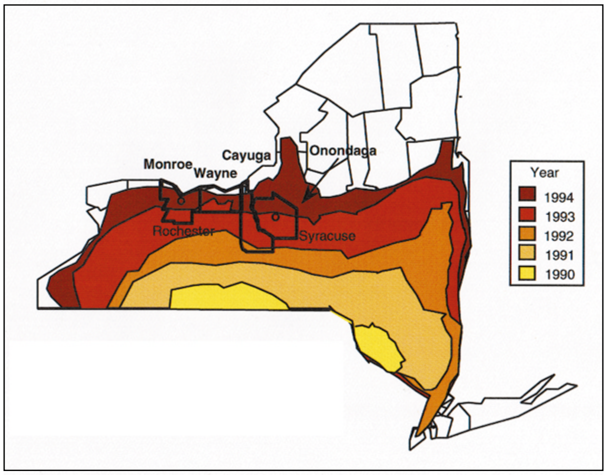

Figure 1. New York State raccoon rabies epizootic progression (1990-94). The raccoon rabies epizootic first affected Monroe, Wayne, Cayuga, and Onondaga Counties between December 1992 and June 1993.

### Data Characteristics and Sources

We considered all human rabies PEP cases reported in 1993 and 1994 for the study area. The PEP capture rate was believed high because local health units were responsible for providing funds for any treatment expenses not covered by health insurance, and a completed, rabies report form was required before reimbursement of the local health unit from state funds.

The New York State Sanitary Code requires physicians to report potential human exposure to rabies and PEP administration to county health departments. We abstracted data from these standardized reports and patient records. Data were grouped by patient demographics, animal characteristics, and exposure details. Exposure source was defined as the suspected- or confirmed-rabid animal that directly or indirectly resulted in one or more potential human exposures to rabies. Direct contact exposure consisted of direct contact (e.g., bite, scratch) or contamination of mucous membranes with potentially infectious material from a rabid animal. Indirect contact consisted of contamination from a fomite (e.g., through racoon saliva on a pet's fur with a pet owner's open wounds or mucous membranes).

### Analyses

Population figures from the 1990 New York State census were used to calculate the incidence of PEP by county, age, and gender (6). Descriptive analyses of data elements were made through queries of Microsoft Access relational database. Each PEP contributed to the denominator of the analyses. Since multiple PEP cases occurred from exposure to a single animal, data for individual animals were also summarized. Chi-square tests were performed with Epi-Info Version 5 software.

### Findings

### PEP Incidence

The annual PEP incidence for the study area increased from <1 case per 100,000 residents in pre-epizootic 1992 to 35 cases in 1993 and 52 cases in 1994. Of 1,173 cases of human rabies PEP in the study areas, 474 were reported in 1993 and 699 cases were reported in 1994. The mean annual incidence of PEP was 32 cases per 100,000 for the urban counties (Monroe and Onondaga; 315 residents per square kilometer) and 123 cases per 100,000 for the two rural counties (Wayne and Cayuga; 51 residents per square kilometer).

### Season

The number of PEP cases peaked in summer to early autumn (Figure 2). During 1993, the highest number of PEP cases occurred approximately 4 to 6 months (August through November) after the invasion of raccoon rabies during March through June 1993; in 1994, the highest number occurred in summer (June through August).

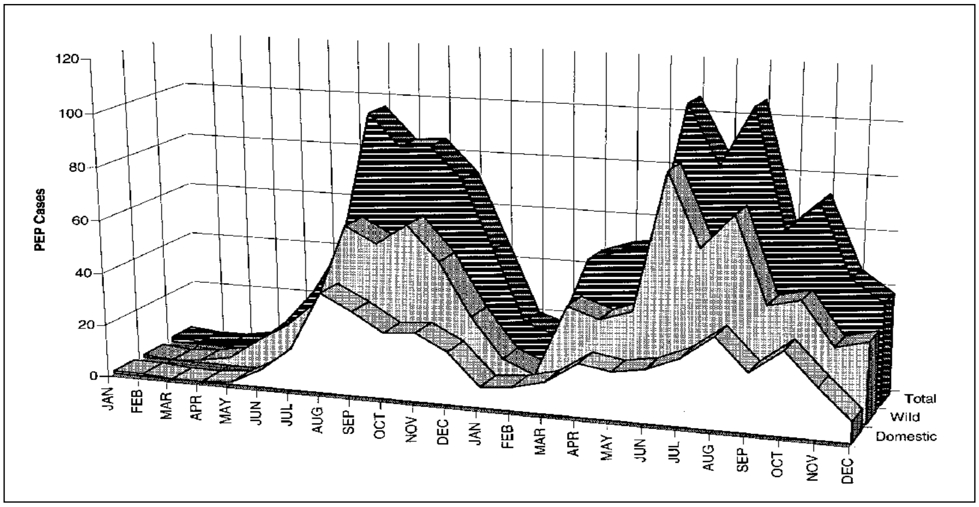

Figure 2. Human rabies postexposure prophylaxis in four New York State counties (Cayuga, Monroe, Onondaga, and Wayne), 1993-1994, by month.

### Gender and Age

Gender and age data were available for 100% and 95% of all patients, respectively. Of 1,173 PEP cases, 642 (55%) were administered to male and 531 (45%) to female patients. The mean annual incidence of PEP in male and female patients was 47 and 38 per 100,000, respectively. The PEP rates were highest in persons 10 to 14 years of age (165 per 100,000) and 35 to 44 years of age (113 per 100,000) (Figure 3). The median age was 29 and 31 years for male and female patients, respectively. No significant relationship was observed between gender and age groups for the study area.

### Exposure Source Species

Exposure to wild animals accounted for 783 (67%) of all PEP cases (Table 1). Among wildlife, raccoons were by far the leading source of exposure, accounting for 589 (75%) of 779 PEP cases due to wildlife exposure. The other sources of wildlife exposure were bats (54 cases), skunks (35 cases), foxes (28 cases), white-tailed deer (13 cases), woodchucks (12 cases), small rodents (9 cases), sika deer (4 cases), and other wild species (39 cases). Of 390 domestic animal exposures resulting in PEP, 205 were attributed to cats, 165 to dogs, 12 to cattle, 5 to pet rabbits, and 3 to horses. Among PEP cases resulting from exposure to cats and dogs, 66% and 67%, respectively, were initiated after contact with stray animals unavailable for the recommended 10-day confinement and observation to rule out rabies or euthanasia and testing. Dog exposures were disproportionately higher in urban (137 [18%] of 753) than in rural counties (28 [7%] of 420) ($p < 0.001$) (Table 2). In urban areas, dog exposure was primarily due to stray or unowned dogs (95 [69%] of 137). In rural areas, stray or unowned dogs accounted for 11 (39%) of 28 dog exposures ($p < 0.01$). Only one dog (in a rural county) tested posi-

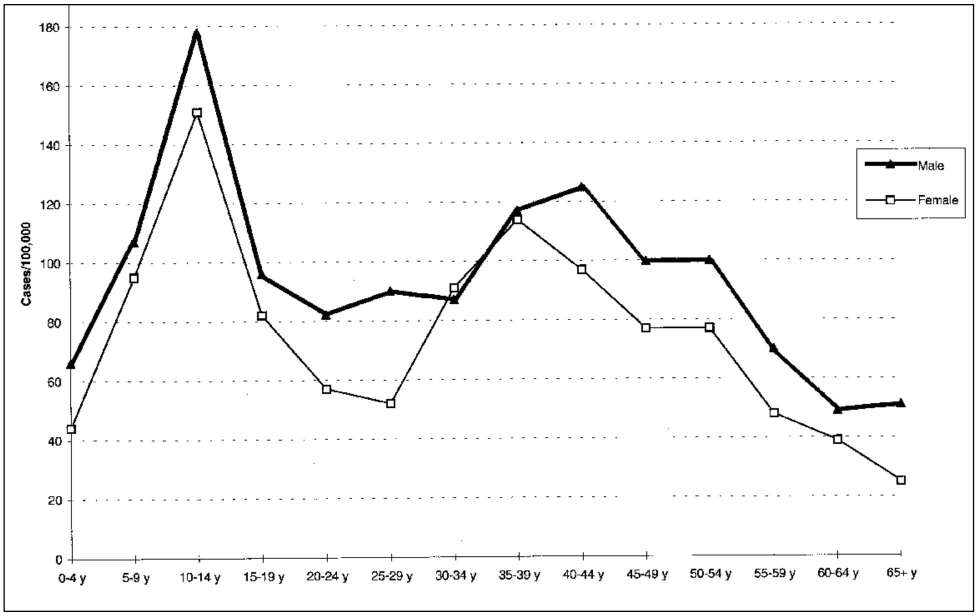

Figure 3. Human rabies postexposure prophylaxis in four New York State counties (Cayuga, Monroe, Onondaga, and Wayne), 1993-94: incidence by gender and age.

Table 1. Human rabies postexposure prophylaxis (PEP), New York State, 1993-94[a]

| Animal source | Bite (N=355) N ( %) | Nonbite (N=818) Direct[b] Scratch N (%) | Saliva N ( %) | NT N (%) | Blood N (%) | Indirect[c] Saliva N (%) | Overall total PEP N (%) |
|---|---|---|---|---|---|---|---|
| Raccoon | 37 (10) | 18 (33) | 44 (29) | 4 (67) | 13 (93) | 472[d] (79) | 589[e] (50) |
| Bat (all species) | 29 (8) | 3 (6) | 12 (8) | 1 (17) | 0 (0) | 9 (2) | 54 (5) |
| Other wild species | 24 (7) | 5 (11) | 21 (14) | 0 (0) | 1 (7) | 89 (15) | 140 (12) |
| All wild species | 90 (25) | 26 (47) | 77 (51) | 5 (84) | 14 (100) | 570 (96) | 783 (67) |
| Cat | 114 (32) | 29 (53) | 41 (28) | 0 (0) | 0 (0) | 21 (4) | 205 (17) |
| Dog | 151 (43) | 0 (0) | 13 (9) | 0 (0) | 0 (0) | 1 (<1) | 165 (14) |
| Other domestic species | 0 (0) | 0 (0) | 19 (13) | 1 (17) | 0 (0) | 0 (0) | 20 (2) |
| All domestic species | 265 (75) | 29 (53) | 73 (49) | 1 (17) | 0 (0) | 22 (4) | 390 (33) |
| Total | 355 (30) | 55 (55) | 150 (13) | 6 (0.5) | 14 (1) | 592 (51) | 1,173 (100) |

[a]Data are from Cayuga, Monroe, Onondaga, and Wayne Counties.
[b]Direct contamination of an open wound or mucous membrane with potentially infectious material such as saliva, nervous tissue (NT), or blood (mixed with other body fluids), from a rabies-suspect or known-rabid animal.
[c]No direct contact with a rabid or suspect-rabid animal. Indirect exposure through possible conveyance of saliva on an animal (i.e., pet dog or cat) or inanimate object resulting in contamination of an open wound or mucous membrane.
[d]$p < 0.001$. More people received PEP after indirect exposure to saliva from raccoons than from any other species (472 PEP cases due to indirect contact with 261 raccoons).
[e]Total PEP cases with raccoon as an exposure source (includes one case with no reported route of exposure).

Table 2. Human rabies postexposure prophylaxis (PEP) in New York State, 1993-94: urban and rural settings[a]

| Animal source | Urban N (%) | Rural N (%) | All four counties N (%) |
|---|---|---|---|
| Dog[b] | 137 (18) | 28 (7) | 165 (14) |
| Cat | 130 (17) | 75 (18) | 205 (17) |
| Other domestic species[c] | 5 (<1) | 15 (4) | 20 (2) |
| All domestic species | 272 (35) | 118 (28) | 390 (33) |
| Raccoon | 341 (45) | 248 (59) | 589 (50) |
| Bat (all species) | 41 (5) | 13 (1) | 54 (5) |
| Striped skunk | 29 (4) | 6 (<1) | 35 (3) |
| Fox 19 (3) | 9 (2) | 28 (2) | |
| Other wild species[d] | 51 (4) | 26 (2) | 77 (7) |
| All wild species | 481 (65) | 302 (72) | 783 (67) |
| Total | 753 | 420 | 1,173 |
| Rate per 100,000 pop. | 32 | 123 | 43 |

[a]Characteristics of human rabies PEP cases reported to the health departments of the two relatively urbanized counties, Onondaga and Monroe, and the two relatively rural counties Cayuga and Wayne, during 1993 and 1994.
[b]$p < 0.00$. Human PEP rates due to dog exposures were significantly higher in urban counties.
[c]Other domestic species include 2 and 3 PEP cases due to cow and horse exposure in the urban counties and 10 and 5 cases due to cow and domestic rabbit exposure in the rural counties, respectively.
[d]Other wild species includes 17, 6, 4, 2, 2, and 1 PEP cases due to an unknown animal type, wild rodent (other than woodchuck), 4 Sika deer (exotic, captive species), opossum, coyote, and mink in the urban counties and 17 and 3 PEP cases due to an unknown animal type and wild rodent (other than woodchuck) in the rural counties, respectively.

tive for rabies in the study area in 1993 and 1994. Of 68 pet cats resulting in human exposure, 61 (90%) were not vaccinated against rabies compared with 14 (24%) of 58 pet dogs ($p < 0.001$).

### Type of Exposure

Of 1,173 PEP cases, 355 (30%) resulted from animal bites and 817 (70%) from nonbite encounters (Table 1). A route of exposure not reported in one case involved a raccoon. Suspected contact with animal saliva (148 cases from direct contact and 594 from indirect contact) was responsible for 742 (91%) of 817 PEP cases due to nonbite exposure; contact with nervous tissue (6 PEP cases) or blood (14 PEP cases) accounted for 2% of cases due to nonbite exposure. Fifty-five (7%) of 817 nonbite exposures were attributable to scratches from 23 cats (responsible for 29 PEP cases), 16 raccoons (18 cases), 3 bats (3 cases), 2 wild rodents (2 cases), and 3 other wild animals (3 cases). Of 355 bite exposures, 265 (75%) involved domestic animals (151 due to 150 dogs, 114 due to 108 cats); 90 (25%) involved bites from wild animals—34 raccoons (responsible for 37 PEP cases), 27 bats (29 cases), 9 rodents (9 cases), and 13 other wild species (15 cases).

### Mode of Contact

Of 1,173 cases, 594 (51%) occurred because of possible indirect contact with a suspect rabid animal; 583 (98%) of 594 occurred after suspected exposure to saliva from a rabid (or suspect rabid) animal on the fur of a nonsuspect dog, cat, or other animal. In 9 (2%) of these cases, PEP was administered after suspected exposure by possibly contaminated fomites including door knobs, traps, arrows, a flashlight, and a wire. Possible indirect exposure to dogs with potentially infectious material on their fur resulted in 507 (85%) PEP cases, while suspected indirect exposure by cats resulted in 70 (12%) cases. Other suspected exposure sources were a horse, rabbit, pet duck, chicken, wild bird, captive exotic sika deer, and a person.

### Group Exposure

Exposure of one person to a suspect rabid animal precipitated 625 (53%) PEP cases; the remaining 548 (47%) occurred after more than one person was exposed to the same suspected animal (Table 3). Exposure of a single person was more likely associated with a bite ($p < 0.001$), while group exposure (involving two or more persons) was more likely associated with nonbites ($p < 0.001$). Wild animal species accounted for most group exposures—with three exceptions. The largest group exposures (involving 12, 13, and 14 people) were associated with the handling of rabid domestic animals (before diagnosis) by veterinary clinic employees.

### Rabies Status

The laboratory diagnosis of rabies in the exposing animal was associated with 540 (46%) of all PEP cases (445 due to wildlife and 95 due to domestic animals). Eighty-nine percent of PEP cases attributed to rabid wildlife involved raccoons. In 88 cases, PEP was initiated after contact with animals eventually proven nonrabid. In 544 cases PEP was administered after contact with animals not tested for rabies. Confirmation of rabies in suspect domestic animals occurred in association with 91 (23%) of 390 PEP cases resulting from exposure to domestic species, including 40 due to 5 pet cats, 23 to 5 stray cats, 13 to 1 pet dog, 5 to a domestic rabbit, 7 to a cow, and 3 to a horse. Conversely, in 88 (8%) of all cases PEP was given after encounters with 81 animals subsequently proven nonrabid (35 due to 33 cats, 32 to 32 dogs, 9 to 8 raccoons, 3 to 3 bats, 5 to 2 skunks, 1 to 1 woodchuck, 1 to 1 squirrel, and 2 to 1 muskrat).

Of 540 cases of PEP associated with animals proven to be rabid, 505 (94%) were due to suspected saliva exposure; 22 (4%) and 13 (2%) involved bites or scratches, respectively. Conversely, 71 (81%) of 88 PEP cases associated with nonrabid animals (i.e., laboratory-confirmed as negative or confined and observed to be healthy) occurred after bite exposures. Of the 544 PEP cases associated with animals of unknown rabies status, 48% were due to bites, 45% to suspected saliva contacts, and 7% to scratches.

Wild animals accounted for 98% of the 690 animals submitted and testing positive for rabies in the study area for 1993-94; 613 were raccoons. If animals testing positive for rabies are used as a surrogate for the true incidence, an

Table 3. Human rabies postexposure prophylaxis (PEP) in New York State, 1993-94: Epidemiologic characteristics[a]

| | Group size | | | | | | | | | | | |
|---|---|---|---|---|---|---|---|---|---|---|---|---|
| | 1 | | 2 | | 3 | | 4 | | 5 | | 6+ | |
| Characteristic | N | % | N | % | N | % | N | % | N | % | N | % |
| Number | 625 | 53 | 180 | 15 | 84 | 7 | 112 | 10 | 60 | 5 | 112 | 10 |
| No. of sources | 625 | 79 | 90 | 11 | 28 | 4 | 28 | 4 | 12 | 2 | 13 | 2 |
| Route of exposure | | | | | | | | | | | | |
| Bite[b] | 328 | 52 | 17 | 9 | 4 | 5 | 3 | 3 | 0 | 0 | 3 | 3 |
| Nonbite | 296 | 47 | 163 | 91 | 80 | 95 | 109 | 97 | 60 | 100 | 109 | 97 |
| Unknown | 1 | 0.2 | 0 | 0 | 0 | 0 | 0 | 0 | 0 | 0 | 0 | 0 |
| Source of exposure | | | | | | | | | | | | |
| Dog or cat | 273 | 44 | 16 | 9 | 3 | 4 | 4 | 4 | 10 | 17 | 64 | 57 |
| Other domestic species | 2 | 0.3 | 4 | 2 | 3 | 4 | 4 | 4 | 5 | 8 | 6 | 5 |
| Raccoon | 235 | 38 | 124 | 69 | 57 | 68 | 96 | 86 | 35 | 58 | 42 | 38 |
| Bat | 39 | 6 | 12 | 7 | 3 | 4 | 0 | 0 | 0 | 0 | 0 | 0 |
| Other wild species | 76 | 12 | 24 | 13 | 18 | 21 | 8 | 7 | 10 | 17 | 0 | 0 |
| Mean age (yr) | 33.4 | | 32.5 | | 24.8 | | 22.5 | | 21.8 | | 26.0 | |

[a]PEP data are from Cayuga, Monroe, Onondaga, and Wayne Counties.
[b]Probability of bite exposure for PEP involving single person vs. group of >1 PEP cases, $p < 0.001$.

approximately 20-fold increase in PEP cases per rabid domestic animal compared with each rabid wild animal, regardless of rural or urban region, is seen (data not shown).

### Provoked Exposures

A provoked exposure was characterized by intentional, human-initiated interaction with a suspect rabid animal. Cases resulting from provoked exposure accounted for 392 (33%) of 1,173 of all PEP cases; 248 (63%) involved domestic animals. Most cases resulting from provoked exposure of domestic animals involved cats (162 [65%] of 248) and less frequently, dogs (62 [25%] of 248). Wild animals accounted for 144 (37%) PEP cases from provoked exposure.

### Time of PEP Initiation

The interval between exposure to suspect rabid animals and initiation of PEP was 0 to 43 days (median 2 days). Bite exposures were associated with no delay in treatment; nonbite exposures were associated with a 3- to 4-day interval ($p < 0.001$).

### PEP Regimen

In 1993 and 1994, postexposure biologic products licensed for use in the United States were rabies vaccine adsorbed, human diploid cell vaccine (Imovax), and human rabies immune globulin (HRIG; Hyperab or Imogam). As recommended by the Advisory Committee on Immunization Practices (ACIP), PEP for the rabies-naive person consists of HRIG (20 IU/kg) on day 0 and five doses of rabies vaccine administered on days 0, 3, 7, 14, and 28 (7). Scheduling information was unavailable for our cases.

Administration of PEP biologic products was recorded as complete in 1,016 (87%) of 1,173 PEP cases. Information regarding completion of the treatment series was not available in 15 cases (1%). Appropriate PEP for preimmunized persons consists of two vaccine doses on days 0 and 3 (7) and was administered to 26 persons, accounting for 2% of all cases. Among preimmunized persons, 17 (65%) of 26 PEP cases occurred after occupational exposures by 11 veterinary staff personnel (including two group exposures to proven rabid cats), four wildlife rehabilitators, one health department employee, and one police officer.

In 54 (5%) instances, PEP was discontinued because of lack of clinical signs in 29 dogs (29 PEP cases) and 23 cats (25 PEP cases) confined for the recommended 10-day observation period. Moreover, 34 (3%) PEP cases were discontinued because of rabies-negative laboratory results in 10 cats (10 PEP cases), 7 raccoons (8 cases), 2 skunks (5 cases), 4 dogs (4 cases), 3 bats (3 cases), 1 muskrat (2 cases), 1 woodchuck (1 case), and 1 squirrel (1 case).

After PEP was initiated, 29 (2%) of 1,173 refused to complete the series; two cited adverse reactions. In nine cases PEP deviated from ACIP recommendations: apparently inadvertent scheduling and administration of six total vaccine doses in four patients and intentional omission of HRIG in the treatment regimen of five patients.

### Adverse Effects

The categories available for characterizing adverse effects on the state rabies report form were none, slight, moderate, severe, or unknown. In 596 (51%) of 1,173 PEP cases, no information was recorded. Of 577 responses, 495 (86%) reported no adverse effects resulting from PEP. Adverse effects were characterized as slight by 67 (12%) persons. Moderate adverse reactions including vomiting, nausea, fever, aches, and weakness were reported by 13 (2%) persons. Serious systemic adverse reactions, recorded as anaphylactic shock and serum sickness, occurred in two (0.2%) persons. Both of these patients had received HRIG; PEP was discontinued after one and two vaccine doses in each case.

### Conclusions

The most important finding of this study was that in most cases PEP was administered because of suspected nonbite, indirect exposure to animal saliva, a route conventionally thought of nearly negligible risk in rabies transmission (7,8). Because of effective PEP, public health personnel and health-care workers are primarily challenged with the assessment of exposure to rabies, rather than with treatment of human cases of the disease. Assessment of nonbite saliva exposures are particularly time-consuming and should consist of a thorough, but nonleading, history-taking that elicits the probability or confirmation of mucous membrane or nonintact skin contact and a realistic assessment of the potential presence of infectious saliva on surfaces or pets. Given the invariably fatal outcome of clinical rabies, the tendency may be to administer PEP, even without clear indication of exposure. This tendency may be unwise—not only for economic reasons, but also because, despite their relative innocuity and high potency, modern rabies biologic products, are not risk-free, nor is their supply unlimited.

The first descriptive study of PEP cases associated with the mid-Atlantic raccoon rabies epizootic during 1982-83 (133 patients) also documented that most PEP cases were due to nonbite exposures; however, these principally involved direct exposure to the suspect rabid animal (1).

A 1980-81 nationwide survey of 5,634 PEP cases found an increased risk for occupational and recreational exposure to animals in a rural setting (9). The absolute mean annual PEP rate described in our report of 43 per 100,000 was nearly 10-fold higher than the rate of 4.7 per 100,000 reported in that study. A rate of 66 per 100,000 was reported from two counties (93 people per square kilometer) in New Jersey at the raccoon rabies epizootic front in 1990 (10,11). The incidence of human rabies PEP in New Jersey and this study exceeded by 10- to 20-fold the rates in areas reporting rabies in skunks (12,13), raccoons (14), and mixed wildlife (9,15). The disparity may be partially explained by regional epizootic versus enzootic status of wildlife rabies and subsequent variations in the comparative intensity of disease in wildlife populations, as well as recent increases in both human and animal population densities and their close association in suburban settings (2). The previous PEP studies involved communities in which rabies had been enzootic in terrestrial wildlife for decades (9,12-16). However, the mid-Atlantic raccoon rabies epizootic comprises the emergence of a terrestrial rabies variant into areas that had, for the most part, been free of terrestrial rabies. The exceptions were sporadic cases of spillover from geographically widespread, but low-level, bat rabies into terrestrial animals and occasional incursions of red fox rabies from Canada into New York, Vermont, and other northern states (2,16).

Previous studies of PEP trends in the United States identified bites from dogs and cats as the most common animal encounter, accounting for 65% to 84% of PEP cases (7,9,12-15). By contrast, only 23% of PEP cases in this study were associated with dog or cat bites. In view of current epidemiologic trends in canine rabies-free areas of the United States, if a biting dog appears clinically normal and can be confined and observed for signs of rabies, the decision to administer PEP may be based on suggestive clinical signs and a prompt diagnostic evaluation that confirms rabies rather than on presumptively initiating PEP. Given that cats are now the leading rabid domestic animal in the United States (17), and more specifically that 12 of the 13 domestic species confirmed rabid from the four-county study area during 1993 and 1994 were cats, rabies vaccinations for cats should become more prevalent. Among exposures to owned domestic pets that resulted in human PEP, 9% of cats (versus 76% of dogs) were vaccinated against rabies. Moreover, most of the encounters with dogs that precipitated PEP in urban counties involved bites from stray dogs, indicating the need for enhanced programs for urban dog control.

The economic impact of a new terrestrial rabies variant is substantial (2,18). In 1994, the New York State Department of Health increased its reimbursement to local health units for mandated rabies control activities from $75,000 to $1,080,000 to assist in the expense associated with human rabies PEP, animal rabies testing (11,896 specimens in 1993), and pet immunization clinics (114 in 1993) (4). Local health units in New York State provide funds for treatment expenses not covered by health insurance. With the cost of rabies biologic products alone exceeding $1,500 per treatment series, an exponential increase in the incidence of PEP, as documented in this study, taxes the public health infrastructure. Moreover, unlike red fox rabies, which periodically reinvades northeastern New York from adjoining areas of Canada and Vermont but then dies out, raccoon rabies is expected to persist in affected areas of New York State, as it has in the southeastern United States for the past 5 decades and in the mid-Atlantic and northeastern states more recently (17).

Control of canine rabies in the United States and other industrialized countries was achieved by eliminating the susceptible reservoir population (through stray dog control and mandatory vaccination) (16). Applications of this concept to wildlife is problematic because of the difficulty in capturing wild animals for vaccination or for applying lethal measures. Population reduction alone is not sufficient to control or eliminate terrestrial wildlife rabies variants over large geographic areas (16). An emerging alternative is oral rabies vaccination of free-ranging reservoir populations, although current methods are still in their infancy and the cost-benefit of such interventions warrants further investigation (10).

During the enzootic raccoon rabies in the southeastern states since the early 1950s or the current mid-Atlantic/northeastern United States epizootic, this variant has not been known to cause human rabies deaths. Yet its potential lethality for humans is supported by ample spillover into other wild animal species (predominantly skunks, but also red foxes, bobcats, and woodchucks) and into domestic animals (predominantly cats, but also dogs, cows, horses, goats, and rabbits). Substantial amounts of infectious rabies virus have been identified in the salivary glands of rabid raccoons (19). No biologic or epidemiologic data suggest unique attenuation or change in virulence of this particular rabies variant that would account for a lack of identified human deaths. Instead, epidemiologic data regarding PEP after suspected exposure to raccoon rabies indicate that PEP frequently is administered even when no exposure has been identified. Also, a bite, scratch, or other exposure, such as gross contamination of an open wound or mucous membrane with moist, infectious material from a small carnivore such as the raccoon, would unlikely be unrecognized or ignored. The apparent liberal administration of effective PEP after known bites, scratches, and other suspected exposures from rabid raccoons may have resulted in complete prevention of human deaths due to this variant of rabies virus associated with raccoons.

Although the Healthy People 2000 goal to reduce PEP is worthwhile, better understanding of the circumstances leading to human exposure and formulating ways to reduce exposure is required to meet this objective. Until then, it will be particularly difficult to reduce PEP during an ever-expanding raccoon rabies epizootic.

### Acknowledgments

We thank the following public health nurses and rabies coordinators for data collection and their generosity and enthusiasm in sharing data: Terri Hogan, Lynn Crane, Linda Thompson, and Hope Albino.

Dr. Wyatt is chair of the University of Rochester School of Medicine and Dentistry's Division of Laboratory Animal Medicine and chief veterinarian at the Seneca Park Zoo in Rochester, New York. His research interests include the epidemiology of zoonoses in the environment and the workplace.

### References

1. Jenkins SR, Winkler WG. Descriptive epidemiology from an epizootic of raccoon rabies in the mid-Atlantic states 1982-1983. Am J Epidemiol 1987;126:429-37.
2. Rupprecht CE, Smith JE, Fekadu M, Childs JE. The ascension of wildlife rabies: a cause for public concern or intervention? Emerg Infect Dis 1995;1:107-14.
3. Centers for Disease Control and Prevention. Raccoon rabies epizootic—United States, 1993. MMWR Morb Mortal Wkly Rep 1994;43:269-73.
4. Hanlon CA, Trimarchi C, Harris-Valente K, Debbie JG. Raccoon rabies in New York State: epizootiology, economics and control. In: Proceedings of the 5th Annual International Meeting. Rabies in the Americas, coping with invading rabies epizootics. Niagara Falls, Canada; 1994 Nov 16-19; p. 16.
5. Public Health Service. Healthy People 2000: national health promotion and disease prevention objectives. Washington: U.S. Department of Health & Human Services; 1991. DHHS publication no. (PHS) 91-50213.
6. U.S. Department of Commerce, Economics and Statistics Administration, Bureau of the Census, 1990. Census of population, general population characteristics. New York: The Department; 1990. CP-1-34.
7. Centers for Disease Control and Prevention. Rabies prevention—United States, 1991. Recommendations of the Immunization Practices Advisory Committee. MMWR Morb Mortal Wkly Rep 1991;40:R-3:1-19.
8. Ashfar A. A review of non-bite transmission of rabies virus infection. British Veterinary Journal 1979;135:142-8.
9. Helmick CG. The epidemiology of human rabies postexposure prophylaxis, 1980-1981. JAMA. 1983;250:1990-6.
10. Uhaa IJ, Dato VM, Sorhage FE. Benefits and costs of an orally absorbed vaccine to control rabies in raccoons. J Am Vet Med Assoc 1992;201:1873-82.
11. Spencer LM. Taking a bite out of rabies. J Am Vet Med Assoc 1994;204:479-84.
12. Schnurrenburger PR, Martin RJ, Meerdink GL, Rose NJ. Epidemiology of human exposure to rabid animals in Illinois. Public Health Rep 1969;84:1078-84.
13. Martin RJ, Schnurrenberger PR, Rose NJ. Epidemiology of rabies vaccinations of persons in Illinois, 1967-68. Public Health Rep 1969;84:1069-77.
14. Currier RW, McCroan JE, Dreesen DW, Winkler WG, Parker RL. Epidemiology of antirabies treatment in Georgia, 1967-71. Public Health Rep 1975;90:435-9.

15. Winkler WG, Kappus KD. Human antirabies treatment in the United States, 1972. Public Health Rep 1979;94:166-71.
16. Baer GM. The natural history of rabies. 2nd ed. Boston: CRC Press; 1991.
17. Krebs JW, Strine TW, Smith JS, Noah DL, Rupprecht CE, Childs JE. Rabies surveillance in the United States during 1995. J Am Vet Med Assoc 1996;209:2031-44.
18. Noah DL, Smith MG, Gotthardt JC, Krebs JW, Green D, Childs JE. Mass human exposure to rabies in New Hampshire: exposures, treatment, and cost. Am J Public Health 1996;96:1149-51.
19. Winkler WG, Shaddock JS, Bowman C. Rabies virus in salivary glands of raccoons (*Procyon lotor*). J Wildl Dis 1985;21:297-8.
